# Endocrine role of bone in the regulation of energy metabolism

**DOI:** 10.1038/s41413-021-00142-4

**Published:** 2021-05-20

**Authors:** Ruoyu Zhou, Qiaoyue Guo, Ye Xiao, Qi Guo, Yan Huang, Changjun Li, Xianghang Luo

**Affiliations:** Department of Endocrinology, Endocrinology Research Center, Xiangya Hospital of Central South University, Changsha, Hunan China

**Keywords:** Multihormonal system disorders, Metabolic syndrome

## Abstract

Bone mainly functions as a supportive framework for the whole body and is the major regulator of calcium homeostasis and hematopoietic function. Recently, an increasing number of studies have characterized the significance of bone as an endocrine organ, suggesting that bone-derived factors regulate local bone metabolism and metabolic functions. In addition, these factors can regulate global energy homeostasis by altering insulin sensitivity, feeding behavior, and adipocyte commitment. These findings may provide a new pathological mechanism for related metabolic diseases or be used in the diagnosis, treatment, and prevention of metabolic diseases such as osteoporosis, obesity, and diabetes mellitus. In this review, we summarize the regulatory effect of bone and bone-derived factors on energy metabolism and discuss directions for future research.

## Introduction

**Fig. 1:**
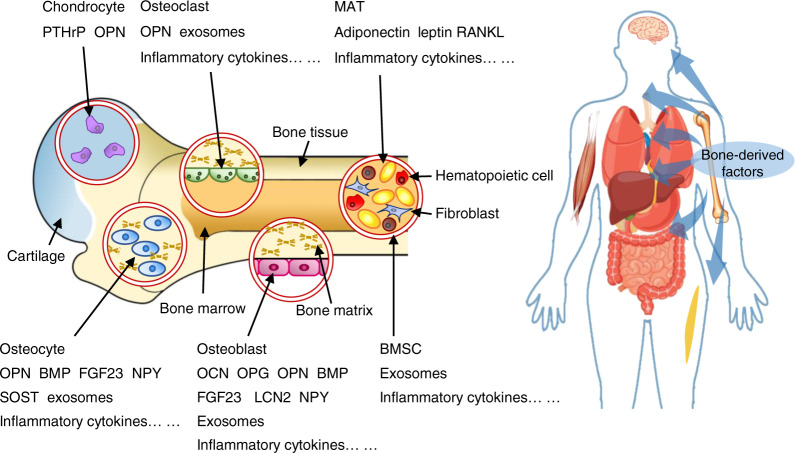
The cells that make up bone mainly include osteocytes, osteoblasts, osteoclasts, and chondrocytes in bone tissue, as well as bone marrow mesenchymal cells and bone marrow adipocytes in the bone marrow cavity. Different cells can produce different endocrine factors, which can enter the blood circulation to regulate multiple organs in the body

The skeleton constitutes up to ~15% of the total human body weight and mainly consists of bone matrix and osteoblasts, osteoclasts, osteocytes and chondrocytes^1^ (Fig. 1). As the fundamental framework of the skeletal system, the bone matrix includes organic matter and inorganic matter. Among them, organic matter contains type I collagen secreted by osteoblasts and a variety of noncollagenous proteins. Inorganic matter, also known as bone mineral, is mainly composed of calcium, phosphorus, magnesium, etc. It serves as an important calcium and phosphorus reservoir in the body.^2^ Osteoblasts can promote mineralization and bone formation by synthesizing osteoids and secreting matrix vesicles. Additionally, osteoclasts can secrete organic acids and proteases to dissolve and absorb bone matrix. Osteocytes, which are the most numerous cells in bone tissue, play an essential role in the regeneration and maintenance of bone matrix. Chondrocytes are the main component of cartilage, and endochondral ossification serves as an important process of bone formation. Bone has hematopoietic function, which is mainly achieved by hematopoietic cells in bone marrow. It is worth mentioning that not only hematopoietic cells but also adipocytes, fibroblasts, and bone marrow mesenchymal stem cells (BMSCs) participate in the regulation of hematopoiesis.^3,4^ However, the role of the skeleton in the pathogenesis of metabolic diseases is poorly understood.

## Bone is an endocrine organ

The role of an endocrine organ is to regulate distant functions through the secretion of a peptide or steroid hormone. Several breakthroughs in bone science in the past few years have elucidated the endocrine role of the skeleton. FGF23 and osteocalcin, which function in a classic endocrine manner, are novel hormones produced by bone cells that control energy balance and mineral homeostasis.

Body homeostasis depends on a dynamic balance of energy metabolism. Once the balance is disrupted, it will lead to the risk of metabolic diseases. Various metabolic organs and tissues, such as the liver, islets, fat, muscle, and skeleton, are involved in energy metabolism. Metabolomic studies have determined the alteration of metabolic pathways during the pathological progression of osteoporosis, which provides strong evidence for the metabolic role of bone in endocrinology. Meanwhile, bone tissue is affected by other circulating hormones, such as adiponectin, leptin, and insulin. Reciprocally, bone-derived hormones exert an effect on energy metabolism throughout the body in return.^5–7^ Recent studies proved that bone cells, including osteoblasts, osteoclasts, BMSCs, and adipocytes, have respective endocrine functions. They can synthesize and secrete a variety of bioactive substances, such as proteins, polypeptides, cytokines, inflammatory factors, adipokines, and exosomes. These bioactive substances regulate bone remodeling by paracrine secretion from bone tissue itself. Additionally, they can be released into the circulation and function by targeting distal organs, thereby affecting the energy metabolism of the whole body.^8–12^

Additional studies have determined that osteocalcin (OCN), which is secreted by osteoblasts, is the first osteoprotein to regulate energy metabolism. It was initially found to promote the proliferation of pancreatic β-cells and insulin secretion,^13^ and later, its other functions in energy regulation were found, including its effects on intestinal epithelial cells, adipocytes, and hepatocytes.^14–16^

In this review, we summarized the bone-derived factors that regulate body energy homeostasis discovered in recent years and offered insights into the mechanisms of how these factors function in the interaction between bone metabolism and energy metabolism (Fig. 1). Concomitantly, this paper also introduces possible directions and challenges in current research, which would be helpful for further investigations.

## Factors secreted by bone cells

Bone tissue harbors several types of specialized bone cells (osteocytes, osteoblasts, osteoclasts, and chondrocytes) that can secrete factors regulating energy metabolism throughout the body (Table 1). Interestingly, some of those factors, such as Lcn2 and sclerostin, are only produced by one type of cell, while others, such as osteopontin (OPN), can be secreted by various types of cells.

**Table 1:** Signaling pathways in energy metabolism regulated by factors secreted by bone tissue cells

Bone-derived factors	Target cells	Signaling pathway	Effect	References
OCN	Islet β-cell	PLC/PKC/Ras/MEK↑→Kv↓→ Ca^2+^↑	Promote β-cell proliferation and insulin secretion	^29,31,34^
		srebp1c/ChREBP↑		
		NF-κB↓→ ER stress↓		
	Adipocyte	Rap1↑→ CREB/PPARγ↑→ ADPN/IL-10↑, TNFα/IL-6↓	Promote fatty acid oxidation and thermogenesis	^39,42,43^
		GULT4↑		
		TCF7↑→UCP1/prdm16↑		
	Hepatocyte	JNK↓, Nrf2↑	Promote hepatic fibrosis and NAFLD	^14^
	Intestinal epithelial cell	GLP-1↑	Promote insulin secretion	^33^
	Muscle cell	AS160 phosphorylation↑	Promote glucose uptake	^27,28^
	Vascular endothelial cell	ER stress, apoptosis↓	Promote insulin signaling	^35,36^
OPG	Islet β-cell	RANK-RANKL↑	Promote β-cell proliferation	^63^
		TRAIL-TRAILR↓, IL-1β↓	Inhibit apoptosis	^73^
OPN	Hepatocyte	FAK/AKT↑→CYP7A1↓	Promote cholesterol formation	^97^
		TNF-α/TGF-β↑, IL-10↓	Promote inflammation	^95,96^
		STAT3↑	Affect gluconeogenesis	^94^
	Islet β-cell	iNOS/NO/NF-κB↓	Decrease cell death	^99^
		Calcium homeostasis	Protect ER	^101^
	Adipocyte	JNK/ERK↓	Promote insulin resistance	^98^
BMP-2, BMP-4	Mesenchymal stem cells	LOX/PPARγ↑	Initiate adipogenesis	^130,133^
BMP-4	Islet β-cell	PKC-θ↑→ IRS-1↓	Promote insulin resistance	^141^
BMP-3b	Adipocyte	PPARγ↓	Reduce transfer of fatty acids	^131^
BMP-8b	Adipocyte	CREB/P38/MAPK/Ucp1↑	Promote thermogenesis	^137^
BMP-7	Hepatocyte	FoxO1↑	Promote glucose uptake	^141^
BMP-9	Hepatocyte	LXRE1↓→SREBP-1c↓	Promote glucose tolerance	^144^
MP-2, BMP-6, BMP-7	Muscle cell, adipocyte	GLUT4 on plasma membrane↑	Promote glucose uptake	^143^
Lcn2	Adipocyte	p38MAPK/PGC/1α-UCP1↑	Thermogenesis	^360^
		IL-6↑, PPARγ/APN↓→GLUT1/GLUT4↓	Decrease glucose intake	^184^
	Hypothalamus	PVN neurons MC4R↑	Increase food intake	^179^

### Osteocalcin

**Fig. 2:**
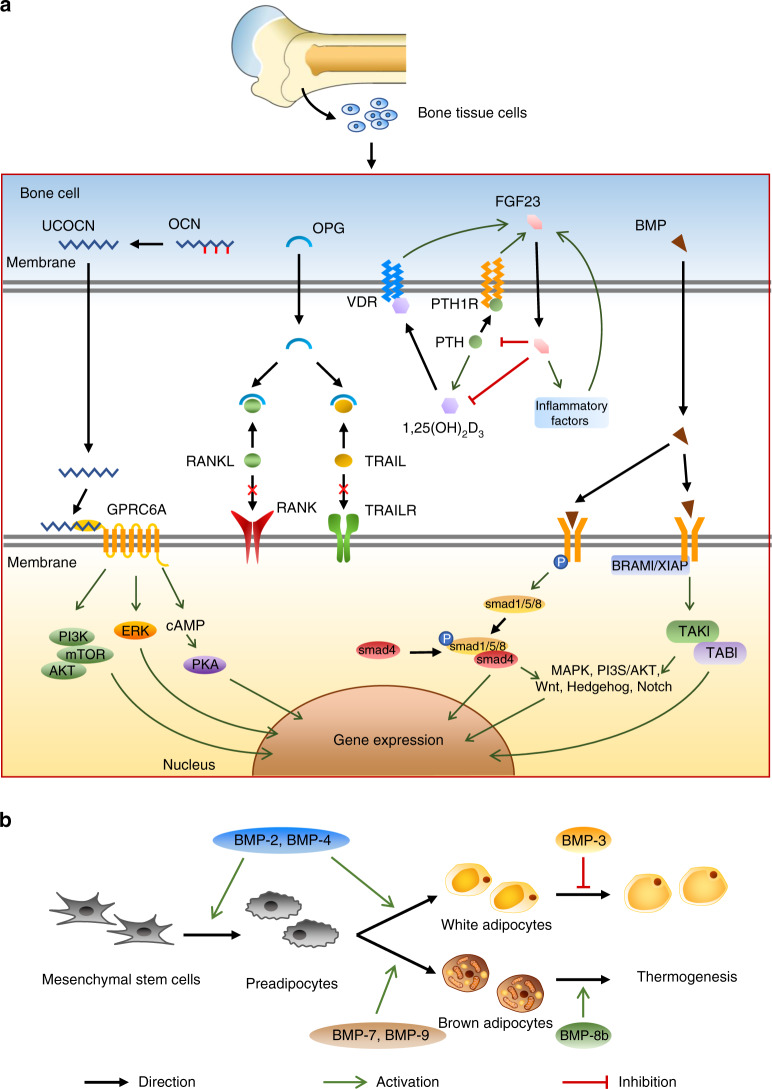
Metabolic regulation of factors derived from cells in bone tissue. **a** OCN can be decarboxylated in osteoblasts, transformed into unOCN, and secreted into the blood. It can primarily bind to the GPRC6A receptor on the cell membrane and activate downstream PI3K/mTOR/Akt signals to affect gene expression. OPG, as a competitive ligand of RANK and TRAIL receptors, can combine with RANK and TRAIL to reduce the distribution of RANK/RANKL and TRAILR/TRAIL on the cell membrane and further affect gene expression. FGF23 can regulate energy metabolism by forming a feedback regulation loop with 1,25(OH)_2_D_3_ and PTH. BMP is mainly derived from osteocytes. Different kinds of BMPs can combine with type I and type II serine/threonine kinase receptors on the cell membrane and can interfere with important downstream pathways, such as MAPK, PI3K/Akt, Wnt, hedgehog, and notch, through Smad protein-dependent or Smad-independent pathways, playing a role in regulating metabolism. **b** The BMP family influences the key steps of fat production and preadipocyte differentiation into WAT and BAT and regulates the function of adipocytes

Osteocalcin (OCN) is a straight-chain polypeptide composed of 46–50 amino acid residues secreted by mature osteoblasts.^17^ After posttranslational modification, the 3 vitamin-K-dependent glutamic acid residues of OCN carboxylate form a γ-carboxy glutamic acid (gla) residue, which is also known as bone glutamic acid protein (gla protein) or γ-carboxylated glutamic acid protein.^18^ Due to the high affinity to mineral ions, the gla residue promotes the storage of carboxylated osteocalcin in bone. Once released from the bone matrix, carboxylated osteocalcin can regulate bone resorption and remodeling by promoting the activity of osteoclasts. However, the bone phenotype varies among different studies; thus, the mechanisms and functions of carboxylated osteocalcin in bone formation/resorption are still controversial.^19–22^ Interestingly, researchers found that OCN knockout mice have higher blood sugar and increased visceral fat.^13,23^ The embryonic stem cell phosphatase (ESP) gene encodes tyrosine phosphatase (OST PTP), which is only expressed in osteoblasts and Sertoli cells of the testis. Lee et al. found that in an ESP gene knockout mouse model, the concentration of circulating OCN was elevated, which was accompanied by an improvement in insulin sensitivity.^17^ These results suggest that OCN can be secreted or released into peripheral circulation in the form of uncarboxylated osteocalcin (unOCN)^24,25^ and promotes glucose uptake, participates in insulin signal transduction, and thus regulates energy metabolism in the whole body^26–28^ (Fig. 2a).

A large number of in vivo and in vitro studies have proven the role of unOCN in the regulation of glucose metabolism. Studies have confirmed that unOCN directly inhibits the expression of the srebp1c and ChREBP genes in islet β-cells, thereby promoting β-cell proliferation.^29,30^ Moreover, unOCN activates the PLC/PKC/Ras/MEK pathway and inhibits kV channels to increase intracellular calcium levels, which plays a vital role in the exocytosis of islet cells.^31^ UnOCN can also promote insulin secretion by regulating other hormones. In intestinal epithelial cells, unOCN has been shown to increase the expression of the insulin stimulating protein glucagon-like peptide-1 (GLP-1) gene.^15,32,33^ In nonislet tissues, unOCN can also increase glucose utilization and improve cell sensitivity to insulin. It has been proven that unOCN can alleviate endoplasmic reticulum stress by activating the PI3K/AKT/NF-κB signaling pathway, thus improving insulin resistance of adipocytes, myocytes, vascular endothelial cells, etc.^34–38^ Interestingly, when NF-κB is activated, unOCN can interfere with NF-κB to prevent further inflammatory response expansion. Both a decrease in the proinflammatory cytokines tumor necrosis factor-α (TNF-α) and IL-6 and an increase in the secretion of the anti-inflammatory cytokine IL-10 slowed systemic inflammation and insulin resistance in mice. At the same time, enhanced expression of slc2a4/GLUT4 was also observed in these mice. As a downstream factor of the NF-κB pathway, slc2a4/GLUT4 mediates insulin-stimulated glucose uptake, which increases glucose utilization.^26–28,39^ In addition to the significant regulatory role of unOCN in systemic insulin signaling, insulin may, in turn, bind to the insulin receptor (IR) on osteoblasts. Thus, it activates insulin signaling in osteoblasts to promote bone remodeling by accelerating the decarboxylation of OCN and increasing circulating unOCN, which indicates a feedforward regulation loop between OCN and insulin.^40,41^

In addition to the regulatory role of insulin metabolism, unOCN can also affect lipid metabolism. unOCN activates the small GTPase Rap1 by expressing cAMP-response element binding protein (CREB) and peroxisome proliferator-activated receptor γ (PPARγ). Consequently, this effect results in adiponectin upregulation, a protein that controls glucose homeostasis and fatty acid oxidation.^16,39,42^ Another metabolic function of unOCN is thermogenesis, which can be detected in brown adipocytes. Upregulation of the T cell factor 7 (TCF7) and PR domain containing 16 (PRDM16) genes and uncoupling protein 1 (UCP1) facilitates the outcome.^43,44^

It is worth noting that unOCN can activate nuclear factor erythroid-2-related factor 2 (Nrf2) and inhibit c-Jun N-terminal kinase (JNK). Among them, Nrf2 is a key regulatory molecule that alleviates lipid peroxidation and oxidative stress. Disorders of Nrf2 trigger liver fibrosis and nonalcoholic fatty liver disease (NAFLD). Moreover, inhibition of the downstream factor JNK, which mediates insulin resistance, slows the occurrence of NAFLD.^13,14,45^ This provides evidence that unOCN can regulate liver metabolism through these two factors (Fig. 2a).

Current studies have indicated that unOCN may be related to the G protein-coupled, family C, group 6, member A (GPRC6A) receptor. GPRC6A receptors are widely distributed and can be activated by various ligands,^46,47^ such as hexapeptide (OCN-6a-c), derived from the C-terminus of OCN. OCN-6a-c acts as a direct ligand of the GPRC6A receptor by binding to GPRC6A.^29,48^ Many experiments have proven that deletion of the GPRC6A gene can eliminate the effect of unOCN on β-cells and adipocytes. This was evidenced by GPRC6A β-cell-cko mice, which exhibited lower circulating insulin levels and impaired glucose tolerance.^29^ Further studies have shown that unOCN activates ERK and cAMP second messenger pathways in GPRC6A-expressing cells in a dose-dependent manner but not in GPRC6A knockout cells.^49^ These results suggest that unOCN regulates energy metabolism by binding to the GPRC6A receptor and activating a series of downstream signal transduction pathways, such as ERK, cAMP, PI3K/Akt/mTOR, and AMPK.^48,50–53^

However, the regulation of unOCN on energy metabolism is still controversial. Recently, Cassandra reported that they did not find an effect of unOCN on blood glucose and insulin levels in OCN-KO mice.^54^ One of the possible reasons is that OCN impacts the internal environment to affect the regulation of some critical signaling pathways. For example, the PI3K pathway is affected by inflammation. In addition, exercise can change the state of the body and adjust the effect of OCN on related tissues. When studying the impact of OCN on skeletal muscle, it was found that the experimental result was closely associated with insulin concentration and muscle movement. Carboxylation of OCN is vitamin-K-dependent. This finding suggests that vitamin K may be an essential regulator affecting osteocalcin function in the human body, especially during bone formation.^17,55^ Another possible explanation is the differences in race and age between the studied subjects.^56,57^ Similarly, dose-dependent activation of GPRC6A induced by unOCN indicates that the concentration of unOCN affects the experimental results. However, these conclusions are conjectures because cross-sectional studies are not enough to explain the causal relationship between osteocalcin and energy metabolism in the human body. Energy homeostasis in the internal environment requires multiple, complex series of factors, and further research is needed in this field.

### Osteoprotegerin

Osteoprotegerin (OPG) is a member of the TNF receptor superfamily expressed in bone, lung, kidney, the cardiovascular system, etc.^58,59^ It has been determined that OPG/receptor activator of NF-κB (RANK)/RANK ligand (RANKL) is an indispensable signaling network for maintaining and regulating bone homeostasis. In bone tissue, OPG inhibits the function of osteoclasts by combining with RANKL and then prevents excessive bone absorption.^60–62^

This regulatory pathway also plays a key role in energy metabolism. For instance, in islet cells, RANK/RANKL is a signal that inhibits the proliferation of β-cells. The phosphorylation of CREB and glycogen synthetase kinase-3 was suppressed by RANK. Then, it can be detected by OPG-treated cells, which hints that OPG can promote the proliferation of β-cells by restraining the RANK/RANKL pathway.^63,64^ Interestingly, epidemiological studies have found an apparent increase in OPG serum concentrations in patients with metabolic syndrome, accompanied by an increase in inflammatory marker C-reactive protein and insulin resistance,^65^ as well as a parallel increase in visceral fat.^66,67^ Similarly, OPG, soluble RANKL, insulin resistance markers, and C-reactive protein were higher in the prediabetic group than in the control group,^68^ and higher OPG levels were also found in obese adolescents.^69,70^

In addition to the OPG/RANK/RANKL signaling system, OPG has another ligand called tumor necrosis factor-related apoptosis-inducing ligand (TRAIL). OPG, as the “fifth receptor” of TRAIL, can competitively inhibit TRAIL and its death receptors, trail-r1 and-r2, thus inhibiting cell apoptosis. Scientists discovered the expression of TRAIL and its receptors in human pancreatic β-cells.^71^ The activation of TRAIL signaling can kill normal pancreatic β-cells, which may contribute to the pathogenesis of type 1 diabetes.^72^ Some studies have certified that the OPG/TRAIL ratio is significantly decreased in the vascular wall of diabetic rats,^73^ suggesting that OPG may regulate energy metabolism through the pathway. In addition, inhibiting the activation of IL-1β signaling by p38 MAPK is a new mechanism by which OPG affects pancreatic β-cells. IL-1β-induced β-cell death requires sustained p38 MAPK activation, which is abolished by OPG.^74–76^ Apart from this, a manifest upregulation of osteocalcin expression and insulin sensitivity can be detected in OPG knockout mice. Therefore, OPG may also regulate bone resorption and glucose metabolism by affecting the secretion of OCN^77^ (Fig. 2a).

Some studies have shown that the level of serum OPG is associated with metabolic diseases, such as NAFLD.^78–80^ However, the relationship between the OPG or OPG/RANK/RANKL signaling system and metabolic disorders is still unclear. It was found that the increased OPG level in type 2 diabetes patients is not due to the onset of diabetes but the progression of disease.^81^ However, OPG and RANKL levels are not related to the risk of type 2 diabetes.^82^ Although osteoblasts secrete large amounts of OPG, the extensive expression of OPG in other organs makes it hard to determine whether bone-derived OPG affects β-cells directly. However, there are still some interesting findings on the OPG/RANK/RANKL signaling system. In addition to osteoblasts, BMSCs and chondrocytes, bone marrow adipocytes can also secrete RANKL.^83,84^ Experiments have shown that in the absence of parathyroid hormone 1 receptor (PTH1R), the expression of RANKL in marrow adipose tissue (MAT) and the content of RANKL in serum were evidently increased. In contrast, it remained unchanged in the thymus and spleen, as well as other tissues that produce RANKL. This result suggests that bone-derived RANKL can be secreted into the circulation to influence distant endocrine organs.^84^

### Osteopontin

Osteopontin (OPN) is a secretory matrix cell protein. In 1985, Heingard et al. first isolated and identified three subtypes of human OPN from salivary protein of bovine bone matrix,^85^ including full-length subtype OPN a, OPN b lacking exon 5 and OPN c lacking exon 4.^86^ OPN is expressed in many cells, including osteoblasts, osteoclasts, chondrocytes, and BMSCs.^87^ It binds to several extracellular receptors, such as integrins (αvβ1, αvβ3, αvβ5, αvβ6, α4β1, α5β1, α8β1, and α9β1) and CD44.^88,89^

In bone tissues, OPN can be secreted by BMSCs and serve as an autocrine cytokine to regulate bone migration, adhesion, and resorption.^90,91^ BMSCs of OPN-null mice are prone to differentiate into adipocytes and exhibit higher body fat content.^92^ In other tissues and organs, the secretion of OPN is mostly related to inflammation. In adipocytes and hepatocytes, OPN primarily enhances inflammatory responses and interrupts glucose homeostasis in cells, which may affect the metabolism of phosphatidylcholine and cholesterol, aggravating the occurrence of nonalcoholic cirrhosis of the liver.^93–98^ However, some other investigations demonstrated that OPN can protect β-cells by reducing the generation of iNOS and preserving Ca^2+^ homeostasis.^99–101^ Existing research in the human population has confirmed the correlation between OPN levels and metabolic diseases.^102–106^ Furthermore, Marciano et al. found that the OPN-encoding gene SPP1 is a susceptibility gene for type 1 diabetes mellitus and regulates the autoimmunity process.^107–109^

Until very recently, studies have demonstrated that OPN secreted by adipocytes, hepatocytes, and macrophages regulates energy metabolism. However, whether bone-derived OPN can perform the same job remains elusive. OPN can regulate the osteogenic or lipogenic differentiation of BMSCs, and a higher body fat content can be detected in OPN global knockout mice. However, research on BMSC-specific deletion of OPN has not yet been reported.^92^ There are only some clinical studies about the effect of bone-derived OPN on metabolism. It is well accepted that a high-fat diet can induce chronic inflammation, while exercise can reduce inflammation. Previous studies showed that the content of OPN increased in mice fed a high-fat diet, and adipose tissue is the principal reservoir of circulating OPN. However, You et al. found that serum OPN in obese teenagers decreased significantly after exercising, while the body fat rate did not change significantly.^110^ These results suggest that tissues other than adipose tissue may contribute more to serum OPN. Since bone tissue is a vital source of OPN and exercise can ameliorate bone metabolism, it is reasonable to consider that dynamic bone metabolism can affect serum OPN levels. Therefore, the mechanism of bone-derived OPN involving global energy metabolism is worthy of deep study.

### Bone morphogenetic protein

Bone morphogenetic protein (BMP) was first isolated from bovine bone. It can promote osteogenesis and chondrogenesis after implantation into bone tissue or extraosseous tissue of mice.^111,112^ It is a subfamily of the TGF-β ligand family.^113^ To date, more than 20 kinds of BMPs have been successfully isolated.^114^ According to their structure, BMP family members can be further divided into several subgroups, including BMP-2/-4, BMP-5/-6/-7/-8, BMP-9/-10, and BMP-12/-13/-14.^115^ BMPs and their receptors are widely distributed in the whole body and exert an essential regulatory role.^116,117^ The most critical regulatory pathway of BMP signaling depends on the phosphorylation of Smad proteins.^118–121^ In addition, BMP signaling also interferes with multiple signaling pathways, including MAPK/PI3K/Akt, Wnt, hedgehog, and notch, and participates in the regulation of various cytokines, such as the IL, INF-γ, and TNF-α. Due to the wide distribution and multiple functions of BMPs, disorder of BMPs may lead to developmental defects or diseases^122–124^ (Fig. 2a).

Bone-derived BMP can be used as an autocrine or paracrine factor to regulate bone itself. For example, in hBMSCs, BMP-2 can promote the expression of inhibitor of differentiation (ID) and runt-related transcription factor 2 (Runx2), thus enabling the development of osteoblasts.^125,126^ BMP also has a profound effect on whole-body metabolism. BMPs can regulate the formation, differentiation, maturation, and biological function of adipocytes^127–140^ (Fig. 2b). Meanwhile, BMPs are involved in insulin secretion of islet β-cells and glucose utilization in different types of cells.^141–144^ Related research in humans has investigated the relationship between circulating BMPs and energy metabolism. This finding demonstrates a positive correlation between fat content and circulating BMP-4 levels in obese individuals.^145^

However, whether the source of circulating BMPs detected in the experiment is bone tissue has not been completely determined. Although osteocytes and osteoblasts are the main sources of BMPs, bone is not the only tissue that secretes BMPs. BMPs are widely expressed in various tissues during the development of embryos.^115^ However, certain specific expressions are manifested when the individual matures. For instance, BMP-3/4/9/10 are located in the lung,^146,147^ BMP-3/7 can be found in nerve cells,^148,149^ and the liver is the primary organ producing BMP-9.^150^ Furthermore, the expression level of BMP-3b in adipocytes is equivalent to that in bone cells.^131^ Therefore, more research is needed to determine whether bone-derived BMPs can be secreted into the circulation and affect energy metabolism in all development stages of the organism.

### Fibroblast growth factors

Fibroblast growth factors (FGFs) regulate multiple processes of growth and development of organisms.^151,152^ Among them, FGF19, FGF21, and FGF23 are called endocrine fibroblast growth factors (eFGFs) because their functions are closely related to metabolic regulation.^153^ eFGFs originate from metabolically active tissue. For example, FGF19 is mainly secreted by the colon,^154^ FGF21 is widely distributed in the liver and pancreas,^155,156^ and FGF23 is a bone-derived protein that is mostly secreted by osteocytes and osteoblasts.^157^ Genetically, it has been proven that serum FGF23 cannot be detected in mice with conditional deletion of the FGF23 gene in osteoblasts and osteocytes.^158^ The FGF23 gene has a high affinity for the FGFR/Klotho coreceptor complex, which is mainly located in the kidney and parathyroid gland.^159,160^ This gene was proven to be closely related to autosomal dominant hypophosphatemic rickets^161^ because it can affect the balance of vitamin D production and the balance between calcium and phosphorus in proximal renal tubules,^12^ which leads to metabolic disorders of bone. Later, it was proven that FGF23 could act directly on bone tissue as an autocrine or paracrine factor. Osteocytes, osteoblasts, and osteoclasts express the FGFR/Klotho coreceptor. Specific knockout of Klotho in osteocytes leads to osteogenic enhancement and an increase in bone mass.^162^ In parallel, related studies have shown that FGF23 can upregulate early growth response genes (EGR) 1 and 2 in osteoblasts and the RANKL/OPG ratio on the osteoclast surface by binding to the coreceptor. Regardless, the activation of the FGFR/Klotho coreceptor may also account for bone remodeling.^163,164^

FGF23 can also act on other tissues to regulate energy metabolism. The most familiar regulatory mechanism is the FGFR/Klotho coreceptor complex. Klotho can bind to membrane receptors and inhibit the phosphorylation of IRs and their intracellular signals, including insulin receptor substrate (IRS) 1 and 2.^165^ Therefore, increased insulin sensitivity and glucose tolerance were observed in FGF23^−/−^ and Klotho^−/−^ mice. The second mechanism is the complex feedback loop between FGF23, vitamin D, and PTH. PTH can promote the expression of FGF23 by increasing the activity of 1,25(OH)_2_D_3_. However, it can also enhance the secretion of FGF23 by acting on PTH1R of osteocytes and activating the cAMP and Wnt pathways.^163^ Conversely, the increased concentration of FGF23 suppresses the expression of PTH and 1,25(OH)_2_D_3_, thus forming a negative feedback loop.^166,167^ The specific role of this regulatory loop in energy metabolism has not yet been clearly studied. However, either PTH or 1,25(OH)_2_D_3_ is the factor regulating energy metabolism, and the imbalance of these factors will inevitably affect the metabolic state. For instance, a study showed that FGF23-null mice had higher insulin sensitivity and lower blood glucose levels, but double mutation of FGF23 and vitamin D receptor (VDR) exacerbated this situation.^166^ Considering the close interrelationship, it is concluded that FGF23 may also regulate energy metabolism through vitamin D (Fig. 2a).

Another mechanism may be related to systemic inflammatory reactions and obesity. FGF23 can reinforce the expression of inflammatory factors in mouse serum and hepatocytes and aggravate liver injury caused by inflammation. Accordingly, the high level of inflammatory factors in the circulation can induce the secretion of FGF23 in bone cells and contribute to a positive feedback cycle between inflammatory factors and FGF23.^168,169^ In hepatocytes, abnormally elevated FGF23 can activate the PLCγ/calcineurin/NFAT signaling pathway and promote the release of more inflammatory factors. However, the physiological concentration of FGF23 may not lead to such changes.^168^ In addition, a study of the elderly Caucasian population showed that serum FGF23 levels were positively correlated with BMI, waist circumference, waist-to-hip ratio, blood lipids, and fat quality.^170^ Because chronic inflammation is one of the causes of obesity, the relationship between FGF23 and inflammatory factors may be a potential mechanism for obesity and lipid metabolism (Fig. 2a).

Another explanation for the energy metabolism regulation function of FGF23 is that FGF23 and FGF19/21 are homologous in structure, and FGF19/21 is involved in maintaining bile acid homeostasis and regulating systemic insulin sensitivity.^171–173^ Therefore, FGF23 may have a similar effect.

### Lcn2

Lcn2, a protein previously thought to be secreted exclusively by adipose tissue and related to obesity, has recently been found to be expressed in osteoblasts. The expression level in osteoblasts is at least 10 times higher than that in white adipose tissue (WAT) or other organs.^174,175^ In addition to acting as an autocrine regulator of adipocytes, Lcn2 also binds to various cell membrane surface receptors, including 1-microglobulin, glycodelin, retinol-binding protein, alpha-1-acid glycoprotein, beta-lactoglobulin, etc.^176,177^

Several lines of evidence have shown that Lcn2 exerts a beneficial effect on the energy metabolism of mice and humans. Its role in the central nervous system has been a significant discovery in recent years. The results showed that the Lcn2 concentration increased threefold in mice fed after fasting, suggesting that Lcn2 can regulate appetite. Lcn2 deficiency is characterized by increased food intake in mice, leading to insulin resistance and obesity. Melanocortin 4 receptor (MC4R), the receptor of Lcn2, controls appetite, body weight, and energy balance in the body.^178,179^ Physically, Lcn2 can penetrate the blood–brain barrier and bind to the MC4R of the PVH nucleus in the hypothalamus.^174^

In the periphery, Lcn2 has also been found to act on metabolism-related tissues. In vitro culture of islet cells showed that Lcn2 could directly act on islet cells and insulin secretion.^174^ In addition, some metabolic effects of Lcn2 have been discovered in adipose tissue. Although there is no evidence supporting that Lcn2 directly binds to the receptor on adipocytes, Lcn2 is a crucial mediator of retinoic acid that mediates the expression of the UCP1 gene and activation of thermogenesis in BAT. Therefore, Lcn2-deficient mice could suffer from decreased circulating retinoic acid levels and have hindered thermogenesis.^180–183^

However, some experimental results have proven the negative effect of Lcn2. GLUT1 and GLUT4 protein levels and glucose uptake in human adipocytes decreased significantly after treatment with rLcn2.^184^ Moreover, Lcn2 knockout mice showed markedly lower fasting blood glucose and higher glucose tolerance than wild-type mice.^185^ Its effect on glucose utilization is probably due to the upregulation of IL-6 gene expression and the reduction in PPARγ and adiponectin.^184^ Further studies in humans also showed that the level of Lcn2 in patients with type 2 diabetes mellitus was significantly higher than that in normal individuals, and its expression was positively correlated with the levels of inflammatory markers such as C-reactive protein, IL-6 and TNF-α.^186^

In general, Lcn2 influences energy metabolism via a complex regulatory network. The complex signaling pathways of Lcn2 and diverse research methods, for example, in vivo versus in vitro experiments and mouse genetic backgrounds, may explain the discrepancy in results from those studies. Another possibility is that Lcn2 secreted by different tissues may have various functions. For example, genome-wide Lcn2 knockout mice showed increased glucose tolerance and unchanged insulin sensitivity,^185^ while osteoblast-specific knockout mice showed decreased glucose tolerance and impaired insulin sensitivity.^174^ Additionally, the different mechanisms and target organs of Lcn2 may account for the different experimental results.

### Sclerostin

Sclerostin (Scl) is a glycoprotein encoded by the SOST gene that is secreted by mature osteocytes.^187^ Several studies have shown that Scl is widely expressed in nearly all tissues and organs, with high expression in bone, especially in osteocytes.^188,189^ Scl can suppress osteoblast and osteoclast activity, which stabilizes the strength and toughness of bone under normal physiological conditions.^187,190^ The lack of Scl will lead to overhardening of bone, and its overexpression will inhibit the formation of bone.^191–193^ The Wnt signaling pathway has emerged as a key regulator of osteogenesis. Scl binds to lipoprotein receptor-related protein 4/5/6 in osteoblasts and osteocytes, a critical coreceptor of the Wnt signaling pathway, and then initiates subsequent signaling cascades.^191,194–196^

Metabolic diseases such as diabetes, obesity, and osteoporosis are usually accompanied by impaired bone formation and low bone mass caused by elevated Scl levels.^197,198^ During this state of metabolic disorder, Scl can not only act on bone tissue but also serve as an endocrine factor that functions in distant organs. Clinical studies by Daniele and Yu et al. found that Scl was associated with fasting insulin levels and insulin resistance in patients with type 2 diabetes.^199,200^ In addition, some other studies in mice with a SOST gene knockout background suggested a decrease in fat content and an increase in insulin sensitivity,^201^ while an increase in Scl levels could promote the formation of beige adipose tissue.^202^ These results indicate that the increase in Scl may not be due to the development of metabolic diseases but may be one of the pathological factors promoting the occurrence of these conditions. However, the specific mechanisms, including genetic drivers behind these findings, are still obscure. The potential role of Scl in whole-body energy metabolism is to combine with Wnt/β-Catenin, which is extensively expressed in the body.

### Neuropeptide Y

Neuropeptide Y (NPY), one of the most abundant neuropeptides in the brain, is a peptide composed of 36 amino acids.^203,204^ In the central nervous system, NPY was initially found to be a robust appetite-stimulating neuropeptide released by agouti-related protein neurons to regulate appetite and energy balance.^205–209^ With a greater understanding of this peptide, it was gradually proven that NPY also had central regulatory effects on circadian rhythm, the cardiovascular system, stress, and anxiety.^205,210^ There are five kinds of NPY receptors (Y1, Y2, Y4, Y5, and Y6) in mammals, which are widely distributed in the central nervous system.^211,212^ An in-depth study of the NPY receptor found its expression in peripheral tissues, including adipose tissue, pancreas, and bone,^213–215^ and the peripheral effect of NPY has received considerable attention. For example, in the pancreas, the activation of the NPY receptor can reduce β-cell apoptosis and hyperglycemia.^216^ The role of NPY in adipose tissue is to promote adipocyte proliferation and adipogenesis.^214^ This suggests that in addition to its secretion in the brain, NPY secreted by peripheral tissues also has profound regulatory functions in the endocrine system.^217,218^

In bone, NPY is secreted by osteoblasts and osteocytes, where it acts on bone tissues through autocrine and paracrine functions.^219^ NPY is generally considered to have no direct effect on bone formation but rescues bone loss in high-fat diet-fed mice.^220^ However, another in vitro study showed that NPY might have proliferative and antiapoptotic effects on BMSCs.^221^ Apart from being secreted from bone, NPY and its receptor are both expressed in many peripheral tissues and play a role in regulating metabolism. A recent study showed that mice lacking NPY had increased fat cells.^222^ However, due to the limited research relating to site-specific knockout of NPY, the specific source of NPY has not been clearly studied.^218^ A study on NPY receptors found that specific knockout of the Y1 receptor in the early osteoblast lineage of mice can lead to increased fasting blood glucose levels and decreased glucose tolerance, which were caused by a decrease in both islet cells and insulin secretion.^223^ Since there is no evidence of NPY binding directly to islet cells, it is possible that NPY might influence the function of osteoblasts, resulting in changes in the metabolism of distant organs. However, the specific mechanism is still unclear.

### PTHrP

Parathyroid hormone-related protein (PTHrP) can be produced by immature chondrocytes and feeds back to promote proliferation and suppress differentiation of chondrocytes. PTHrP can bind to either hypertrophic chondrocytes or hypertrophic mast cells, slowing their proliferation or differentiation.^224–226^ In addition, mice with osteoblast-specific targeted disruption of PTHrP showed decreased expression of PTHrP in bone, indicating that osteoblasts are one of the sources of PTHrP protein.^227^ A robust increase in PTHrP levels can be detected in blood circulation after enhancing the expression of PTHrP in osteoblasts, which indicates that bone-derived PTHrP can be released into the circulation.^228^ This provides a basis for its function of regulating global metabolism.

It was found that the increase in PTHrP gene expression in mouse osteoblasts could be secreted into the blood circulation, improve heat production and glucose tolerance, and decrease fasting blood glucose levels. However, the glucose level in mice was not related to the secretion of insulin and osteocalcin but to lipid metabolism. Further studies have revealed that PTHrP derived from osteoblasts could stimulate the secretion of adiponectin in WAT through the PKA/CAMP and Akt/Fox signaling pathways. As a result, “browning” of WAT and fatty acid oxidation were activated, and the process further reduced glucose production.^228^ Whether bone-derived PTHrP can directly affect β-cells remains to be further studied. Nevertheless, a recent study only found that PTHrP and its receptor are expressed in β-cells, and the overexpression of PTHrP increased the expression of the G1/S cell cycle activator CDK2 and cyclin E, thus promoting the proliferation of islet cells.^229^ The mechanisms by which PTHrP regulates energy metabolism in individuals warrant further exploration. Since PTHrP has been proven to be a safe drug for osteoporosis treatment,^230^ it may have great prospects in treating obesity and diabetes.

## Factors secreted by BMSCs

BMSCs are multipotent stem cells with the potential to differentiate into many different types of cells. Moreover, BMSCs are a crucial part of the bone marrow microenvironment that regulates hematopoiesis. Metabolic disorders such as obesity and diabetes mellitus are prone to inducing adipogenic rather than osteogenic differentiation of BMSCs, which contributes to osteoporosis and bone fracture in patients.^231^ BMSCs have been used in cell transplantation therapy to treat metabolic diseases. In animal experiments and clinical trials, BMSC transplantation promoted the proliferation of islet cells and reduced insulin resistance.^232,233^ On the other hand, BMSCs can secrete some active substances and affect bone metabolism and energy metabolism throughout the body.^234–236^

### Exosomes

Exosomes are extracellular vesicles (EVs), and researchers have focused on structural engineering approaches in recent years to drive regenerative concepts. The diameter of exosomes is between 40 nm and 150 nm, and they contain proteins, RNA, cytokines, etc. Exosomes are released by almost all cell types, including stem cells.^237^ Exosomes can enter the blood circulation where they mediate communication between cells, and they have a broad regulatory effect on the body.^238–241^

In bone tissue, exosomes are secreted by almost all kinds of cells.^242–245^ The components of vesicles are rich in variety, including NF-κB, RANKL, ephrinA2, semaphorin 4D, miR-146a, and miR-214-3p. These exosomes mediate the regulation of different kinds of cells and their functions in bone. For example, osteoclasts can release exosomes containing RANK to suppress osteoclastogenesis.^246^ Conversely, exosomes containing miR-214 are secreted by osteoblasts and inhibit their function.^244,247,248^ Recently, new studies have found that the expression of RNA in exosomes secreted by BMSCs is similar to that of exosomes produced by stem cell-derived adipocytes.^249^ This suggests that the two kinds of exosomes may share similar abilities to regulate whole-body energy metabolism.

**Fig. 3:**
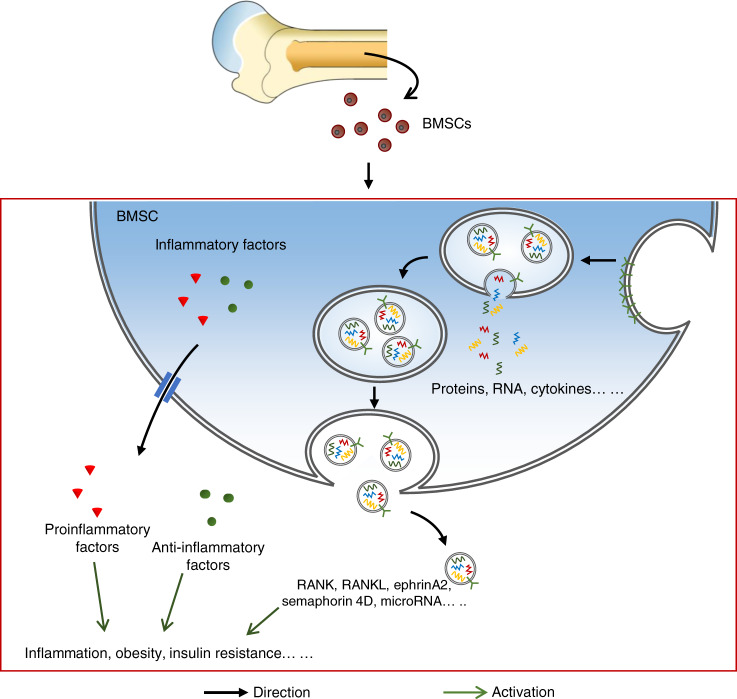
Exosomes and inflammatory factors are mainly secreted by BMSCs. After forming vesicles containing various active substances, exosomes are released into the extracellular matrix. The inflammatory factors released by BMSCs can be divided into proinflammatory factors and anti-inflammatory factors. Exosomes and inflammatory factors can regulate distant target organs, thus leading to an inflammatory response, obesity, insulin resistance, etc

Furthermore, Su et al.^250^ confirmed that BMSCs can exert an indispensable function in the pancreas, liver, and other metabolism-related organs through exosomes. They found that exosomes containing miR-29b-3p secreted by BMSCs increased significantly with age. In parallel, an in vitro study showed that these exosomes can induce aging-related insulin resistance and inhibit insulin signaling activation in 3T3-L1 adipocytes, C2C12 cardiomyocytes, and primary cultured hepatocytes. It is well known that the SIRT1 gene benefits insulin resistance and diabetic status. Further studies showed that the SIRT1 gene serves as the downstream target of miR-29b-3p regulating insulin sensitivity, and miR-2 9b-3p can directly conjugate to the SIRT1 gene and suppress its expression.^250^ In addition, because exosomes contain immunoregulatory factors, they also play a role in repairing liver fibrosis, including reducing collagen accumulation, enhancing liver function, inhibiting inflammation, and promoting liver cell regeneration.^251^ Cytologically, exosomes contain various bioactive substances, which make them attractive potential therapeutic sources. An in vivo experiment demonstrated that the injection of BMSC-derived exosomes into a diabetic mouse model could improve glucose tolerance by suppressing TGF-β/Smad3 signaling via exosomal miRNA, suggesting an important role of exosomes in the treatment of metabolic diseases.^252^ In another study, a mouse model of liver fibrosis was treated with exosomes derived from human BMSCs. The regeneration of hepatocytes was restored, and the expression of PPARγ, Wnt3a, Wnt10b, β-Catenin, WISP1, cyclin D1, α-SMA, and collagen I was inhibited.^251^ Furthermore, mitigation of age-related insulin intolerance was observed by injecting miR-29b-3p inhibitor into the bone marrow cavity of aged mice. Exosome contents can also be used as targets for the treatment of diseases^250^ (Fig. 3).

### Inflammatory cytokines

Chronic low-grade inflammation is associated with the pathogenesis of metabolic syndrome.^253^ It is characterized by abnormal cytokine production, increased acute-phase reactants and other mediators, and activation of inflammatory signaling pathway networks.^254,255^ For example, the activation of inflammation in cells of various organs and tissues can activate inflammatory signaling pathways, including NF-κB and JNK, which interfere with IR signal transduction and lead to insulin resistance.^256^ Inflammatory factors can be divided into proinflammatory factors and anti-inflammatory factors.^257^ Bone can regulate energy metabolism by secreting various inflammatory factors.

IL-6, TGF-α, and IL-1β are common proinflammatory factors that can be expressed by adipocytes.^258^ Inflammatory factors secreted by bone can regulate its growth.^259^ It has been determined that IL-6 secreted by osteoblasts can regulate bone resorption.^260^ However, BMSCs seem to be more capable of producing inflammatory factors than osteoblasts.^253,261^ Many inflammatory factors, such as IL-6, macrophage inflammatory protein-1α (MIP-1α), granulocyte colony stimulating factor and granulocyte macrophage colony stimulating factor, were demonstrated to be secreted by BMSCs.^261^ The higher levels of the chemokines CXCL1 and CXCL2 are involved in osteoclastogenesis and are associated with bone marrow adipocytes.^253,262^ Additionally, microarray analysis showed that inflammatory genes such as IL-6 and TNF-α were highly expressed by bone marrow adipocytes.^263^ However, serum levels of these bone-derived proinflammatory factors are usually low, and few studies target systemic effects. Researchers only found that human bone marrow adipocytes can secrete trace amounts of IL-1β and TNF-α but large quantities of IL-6 during an in vitro study. This implies that bone marrow adipocytes may participate in systemic fat metabolism and the inflammatory response.

IL-10 is a type II cytokine with anti-inflammatory properties.^264^ The anti-inflammatory effect requires its binding with the receptor complexes IL-10Rα and IL-10Rβ, which trigger the activation of signal transducer and activator of transcription 3 (STAT3).^265,266^ IL-10 is also expressed by bone marrow cells, and it can be attached to the receptor IL-10Rα on adipocytes. Compared to bone marrow cell-specific IL-10 knockout mice, global IL-10 gene knockout mice have greater WAT content, higher blood sugar levels, and lower glucose tolerance. We can rationally infer that bone marrow-derived IL-10 is a crucial factor affecting the browning of fat and insulin sensitivity.^267^ However, the role of BMSC-derived inflammatory factors in energy metabolism remains largely uncertain, which provides a potential field for exploration (Fig. 3).^268^

## Factor secreted by MAT

Bone MAT accounts for more than 10% of the total human adipose tissue mass.^269,270^ Adipose tissue in bone marrow can be divided into two main categories. One category is regulated MAT (rMAT) in red bone marrow, which is mainly distributed in areas with activated hematopoietic function. The other is constitutive MAT (cMAT) in the yellow bone marrow, which is concentrated in the distal skeletal region. cMAT contains more unsaturated lipids than rMAT and has a similar structure to WAT.^271,272^ The structural difference between cMAT and rMAT leads to distinct functions. It has been found that MAT in vertebrae of mammals has BAT-like thermogenic characteristics, whereas it often exhibits a WAT-like phenotype in the tibia.^273^ Although studies on the formation and transformation of these two kinds of bone marrow adipocytes and their respective endocrine functions are still insufficient, MAT can indeed secrete various regulatory factors to exert its function.^270^ Among them, RANKL from MAT was recently found to be related to energy metabolism, which has been described previously in our review. This suggests that the role of MAT in bone and systemic energy metabolism cannot be underestimated.

### Adiponectin

Adiponectin is abundant in serum.^274^ It was initially thought to be secreted by WAT,^275^ but later, a large amount of adiponectin expression was also observed in MAT. In fact, the expression of adiponectin in MAT was higher than that in WAT.^276^ Cawthorn et al. confirmed that under calorie restriction, the increased level of serum adiponectin is directly related to the volume of MAT but not WAT.^269^ These findings illustrate that MAT might offer alternative supplementary energy for patients with insufficient WAT.

Adiponectin produces a remarkable effect in regulating adipose metabolism, insulin secretion, and other metabolic pathways. The regulatory effect of adiponectin is achieved by acting on receptors 1 and 2 (AdipoR1 and AdipoR2), which are expressed in various tissues and organs, including skeletal muscle, liver, and pancreas.^277–282^ Adiponectin can activate PPARα, AMPK, and p38 MAPK by interacting with AdipoR,^279,283^ thereby regulating glycolipid metabolism. Additionally, adiponectin can even act on the central nervous system by crossing the blood–brain barrier. The increase in adiponectin in the ventricle can lead to weight loss and an increase in energy consumption, which may be mediated by overexpression of corticotropin-releasing hormone in the hypothalamus.^284^

The effect of adiponectin secreted by MAT on systemic metabolism has not been completely determined. The function of adiponectin secreted by MAT has only been proven in the metabolism of skeletal muscle cells. It can stimulate Ca^2+^ influx and liver kinase B1 activation, contributing to enhanced AMPK activity, PPARγ coactivator-1α expression, and mitochondrial biosynthesis.^269^ It is worth noting that adiponectin is highly expressed in MAT and can be secreted into the circulation. Therefore, adiponectin secreted by MAT may impact the metabolism of distal organs. In addition, insulin can inhibit the gene expression of adiponectin in human MAT, suggesting that MAT may be affected in hyperinsulinemia.^285^

**Fig. 4:**
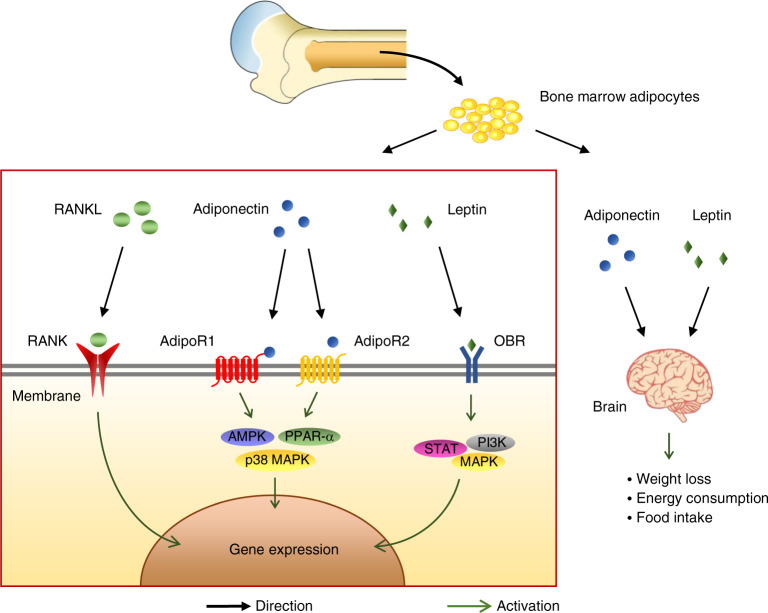
MAT can secrete RANKL, the ligand of RANK. RANKL regulates metabolism by binding with RANK on the cell membrane. In addition, MAT can also secrete the adipokines leptin and adiponectin. Leptin and adiponectin combine with ADIPOR and OBR on the cell membrane of peripheral tissue, regulating cellular sugar metabolism. They also act on the central nervous system, leading to reduced food intake and energy consumption

The transcription and translation of adiponectin found in human osteoblasts was low.^286^ Mature osteoblasts collected from the tibia and femur were quantitatively analyzed by real-time polymerase chain reaction. The results show that it accounts for only 3% of the adiponectin level in human subcutaneous adipose tissue.^286^ Therefore, the role of adiponectin derived from bone tissue cells in energy metabolism is still under debate. (Fig. 4).

### Leptin

Leptin, which is encoded by the obesity gene (Ob) and secreted primarily by WAT, is directly related to obesity. Mutations of the leptin gene lead to obesity.^287^ Most tissues express leptin receptor (ObR), among which the concentration of leptin receptor in the hypothalamic arcuate nucleus, lung, liver, spleen, kidney, and adrenal gland is relatively high.^288^ Leptin conjugates to ObR, thus triggering cascade reactions of the STAT, PI3K, and MAPK signaling pathways.^289^ For example, leptin can act directly on islet cells and inhibit insulin secretion.^290–292^ It can also regulate adipocytes and alter cell sensitivity to insulin^293^ or work on the feeding center of the brain, thereby inhibiting food intake and reducing fat content. At present, the regulatory mechanism of leptin on energy metabolism has been clearly studied.

It was recently discovered that MAT also expresses leptin.^294^ Rosiglitazone treatment can promote browning of adipose tissue in bone marrow and increase leptin expression.^295^ An in vitro study illustrated that the gene expression of leptin could be significantly inhibited by proinflammatory cytokines, hematopoietic cytokines, IL-1β, IL-6, TNF-α, and IFN-γ in human bone marrow adipocytes.^296,297^ Therefore, in the state of systemic inflammation, such as obesity, inflammatory factors can affect the endocrine function of MAT, leading to further deterioration of the inflammatory response. Additionally, leptin expressed in bone MAT can directly act on leptin receptors in bone and affect bone growth by activating FGF23 and regulating the secretion of osteocalcin.^298^ However, there is no clear evidence that leptin from MAT can regulate global metabolism (Fig. 4).

## Circulating endocrine factors affect both bone and energy metabolism

There is a complex relationship between bone and energy metabolism. The main action of circulating endocrine factors is not limited to regulating energy metabolism but affects bone metabolism and remodeling.

### Insulin

Insulin is an important hormone regulating energy metabolism in the human body, and its receptor exists in almost all cells. Insulin promotes glycogen, fat, and protein synthesis by binding to IRs and ultimately lowers blood sugar.^299^ In the skeleton, insulin can activate insulin signaling pathways in osteoblasts and osteoclasts by binding to IR and IRS.^300^ In osteoblasts, it can inhibit Twist2, a Runx2 inhibitor, and thus promote the differentiation of osteoblasts required for normal bone formation.^301^ Fulzele et al. demonstrated in mice that when osteoblasts lack IR, osteoporosis can easily develop after birth.^302^ In osteoclasts, insulin can upregulate the expression of RANK, increase the binding of RANK and RANKL, and ultimately contribute to the production of osteoclasts.^303^ In addition, IRS-1 deficiency inhibits the proliferation of chondrocytes through the PI3K/Akt pathway, which is an adverse factor for bone healing.^304^ Clinical observations have shown that excessive insulin seems to reduce bone turnover and increase bone mineral density. However, it may also lead to bone fragility by increasing cortical osteoporosis or other bone structural defects.^305^

The regulation of bone by insulin can affect whole-body energy metabolism. Insulin can promote osteoblast differentiation and glucose uptake by increasing the expression of Glut4 on the cell membrane. The absence of this pathway will lead to peripheral insulin resistance, which is mainly manifested by an increase in systemic insulin levels and a decrease in insulin sensitivity. However, no significant phenotypes were found in skeletal muscle cells and adipocytes. Therefore, the decrease in insulin absorption by bone may lead to systemic insulin resistance, which proves the influence of insulin and bone on whole-body energy metabolism from another aspect.^306^ On the other hand, insulin resistance in osteoblasts leads to decreased circulating osteocalcin levels, reducing insulin secretion and insulin sensitivity in skeletal muscle, thus leading to systemic glucose intolerance.^307,308^

### Vitamin D

Vitamin D is a steroid hormone that plays a vital role in maintaining bone metabolism and calcium homeostasis.^309^ As an active form of vitamin D, 1,25(OH)_2_D_3_ can promote calcium absorption^310^ and regulate calcium reabsorption^311^ through conjugation to the VDR. On the other hand, when the serum calcium level declines, VDR in osteoblasts can enhance bone resorption, thereby mobilizing bone calcium into the blood to maintain serum calcium homeostasis.^312^ In recent years, it has been gradually found that vitamin D has other physiological effects. The relationship between vitamin D and metabolic diseases such as obesity, diabetes, and NAFLD is a trend in research. Adipocytes are the main storage sites of vitamin D and express active vitamin D and VDR.^313,314^ Meanwhile, vitamin D can regulate adipose formation from multiple perspectives, and its effect depends on the level of vitamin D, the type of adipocytes and the differentiation level.^315–318^ It was also found that patients with NAFLD were more likely to suffer from decreased bone mineral density and vitamin D deficiency. Vitamin D supplementation can reduce the risk of elevated blood glucose and insulin resistance.^319,320^ This suggests that bone may be a bridge for vitamin D to regulate energy metabolism.

### Parathyroid hormone

Parathyroid hormone (PTH) can directly affect bone and kidney to regulate calcium and phosphorus metabolism. One of the key mechanisms of PTH in regulating calcium homeostasis is stimulating bone remodeling. It not only promotes bone resorption and mobilizes bone calcium into blood but also promotes osteogenesis and ameliorates osteoporosis. PTH1R is expressed on the surface of osteoblasts and many bone cells.^84^ The catabolic function of PTH is mainly achieved indirectly by acting on osteoclasts through the OPG-RANKL-RANK pathway.^321^ In contrast, the anabolic effect is conducted directly by working on PTH1R in osteoblasts and osteoclasts, further promoting the differentiation of precursor cells^322,323^ and inhibiting the apoptosis of mature cells and the expression of Scl.^324–326^ PTH can also enhance aerobic glycolysis in osteoblasts by promoting the transduction of the insulin-like growth factor signaling pathway, thereby enhancing the synthesis function of bone in mice.^327^

In addition to regulating systemic calcium and phosphorus metabolism, PTH also has some effects on energy metabolism, which may be related to the level of vitamin D. It was found that the level of blood sugar increased significantly after parathyroidectomy and decreased after PTH administration, while the secretion of insulin remained unchanged.^328^ Kimura et al. also demonstrated that PTH could reduce the blood sugar level in obese type 2 diabetic rats without changing the serum insulin level.^329^ However, PTH can affect insulin sensitivity. One piece of evidence is that the insulin sensitivity index is negatively correlated with plasma PTH levels.^330^ In addition, excessive PTH leads to disorders in lipid metabolism. PTH induces lipolysis of adipocytes by activating the cAMP-PKA pathway, leading to increased serum cholesterol and triglyceride concentrations in mice.^331–333^

### Estrogen

In addition to reproductive function, estrogen also regulates bone metabolism and energy metabolism. Lack of estrogen increases the risk of metabolic syndromes such as obesity and type 2 diabetes mellitus.^334,335^ The main estrogen receptors are ERα and ERβ,^336^ which are distributed in multiple tissues of the body. In the central nervous system, activation of ERs in the ventromedial hypothalamus and arcuate nucleus can control dietary intake.^337–339^ In the periphery, estrogen can act on ERs to increase insulin sensitivity in adipose tissue, skeletal muscle, and liver.^340–342^ For example, estrogen can inhibit adipogenesis^341^ and reduce the expression of lipoprotein lipase, an important regulator of lipoprotein metabolism.^340^ Moreover, an experiment showed that ER agonist therapy increased the expression of GLUT4 and glucose uptake in rat skeletal muscle.^342^ The role of estrogen in bone has also been affirmed. Postmenopausal estrogen deficiency can lead to osteoporosis, which is due to estrogen inducing osteoclast apoptosis through ERs and protecting bone through the inflammatory mediators RANKL and Scl.^343–346^

## Prospects

It has been known for a long time that skeletal health is closely related to the overall metabolism of the individual. For example, people with diabetes have a greater risk of developing osteoporosis than normal individuals,^347,348^ and a low body mass index causes bone loss.^349,350^ The presence of insulin, adiponectin and leptin-related receptors in bone proves that the energy metabolism of the whole body has a direct impact on bone.^307,351,352^ However, bone tissue’s constant turnover suggests active energy generation and consumption in osteogenesis and bone resorption, which will inevitably affect whole-body energy metabolism. Later, through the discovery of osteocalcin, the first bone-derived factor that regulates energy metabolism, researchers found that bone could also regulate energy metabolism throughout the body. Some findings showed that bone-derived factors, such as Lcn2 secreted by osteoblasts and adiponectin and leptin secreted by MAT, could cross the blood–brain barrier and act on the central nervous system, which is one of the discoveries in recent years. Further study on bone-derived factors and their interactions will help to understand the fundamentals of the central nervous system for energy balance. Bone-derived exosomes are also a new research area in recent years and have been found to regulate bone itself and other tissues.^245^ However, due to the universality of exosomes and the diversity of their contents, more functions of bone-derived exosomes need to be explored.

The effect of bone-derived factors on the regulation of energy metabolism-related organs is complicated. This may be due to the different distributions and proportions of receptors on the surfaces of other cells so that these factors have more than one regulatory pathway. Moreover, sometimes the results of experiments on animals and cell cultures in vitro may be different from those obtained from human subjects, indicating that the human body has a more complex internal environment, which impacts the results. Bone-derived factors may be affected by a variety of factors in the processes involved in secretion, circulation and function, including genetic background diversity, age and health status of the human body. For example, decarboxylation of OCN is vitamin K dependent, so the vitamin K concentration in the human body will affect the physiological function of osteocalcin.

One of the key points in studying the regulation of bone-derived factors on energy metabolism is to confirm whether the change in the levels of those factors is the cause but not the result of altered energy metabolism. Because of the close relationship between bone metabolism and whole-body energy metabolism, the levels of factors secreted by bone are likely to be determinants of energy homeostasis and metabolism. Second, it is of interest to determine whether those factors that play a crucial role in other tissues and organs are from bone. This could be easily determined for some factors, such as osteocalcin and FGF23, which are mostly secreted by bone cells. For other factors expressed in multiple tissues and organs, most of them need to be assessed through genetic engineering techniques by using animal models (Table 2). However, at present, not all bone-derived factors may possess the necessary gene-specific knockout animal models applicable to energy metabolism regulation experiments, such as BMP and OPN. Another reason is that although some autocrine factors in bone cannot be secreted into the blood circulation to act on other organs, they can indirectly affect systemic metabolism by regulating bone metabolism, which should not be ignored.

**Table 2:** Conditional gene knockout mice used for studies of energy metabolism and bone metabolism

Bone-derived factors	Transgenic mice	Metabolic changes	Main conclusions	References
OCN	Osteoblast-specific ESP deficient mice (Col1a1-Cre)	Increased circulating glucose level; decreased insulin secretion and sensitivity l; impaired glucose tolerance; insulin resistance; decreased circulating adiponectin level; decreased energy expenditure.	OCN secreted by osteoblasts can (1) promote the proliferation of β-cells and increase the secretion of insulin, (2) enhance the expression of adiponectin in adipocytes and improve the insulin sensitivity.	^23^
OPG	Osteoblast-specific OPG overexpression mice (Osterix-Cre)	Increased total body fat amount in adipose tissue	The high expression of OPG in osteoblasts saved the increased fat reduction and energy consumption caused by β-catenin knockout.	^361^
BMP	Chondrocyte specific Bmp2 deficient mice (Col2-Cre)		BMP2 can promote fracture healing.	^362^
FGF23	Osteoblast and osteocyte specific FGF23 deficient mice (Col2.3-cre, Dmp1-cre)	Increased circulating phosphate level	FGF23 secreted by osteoblasts and osteocytes can reduce the serum phosphorus level.	^158^
FGF23	Global FGF23 knockout mice (FGF23^−/−^)	Improved glucose tolerance; hypoglycemia; decreased body weight	(1) FGF23 deficiency can lead to the increase in basal blood glucose, peripheral insulin sensitivity, and decrease in fat content in mice; (2) the effect of FGF23 on blood glucose, insulin, and the fat content in mice is related to vitamin D signaling.	^238^
Lcn2	Osteoblast-specific Lcn2-deficient mice (Col1a1-Cre)	Impaired glucose tolerance; insulin resistance	Lcn2 secreted by osteoblasts can combine with MC4R in PVN neurons of the hypothalamus, to (1) suppress appetite; (2) increase insulin sensitivity and glucose tolerance.	^174^
Scl	BMSC, osteoblast, osteocyte, chondrocyte specific SOST deficient mice (Prx1-Cre, Col1-Cre, Dmp1-Cre, ColX-Cre)	Increased bone mass	Loss of SOST in limb mesenchyme cells leads to a significant increase in bone mass.	^363^
PTHrP	Osteoblast-specific PTHrP deficient mice (ColI-Cre)	Decreased bone mineral density; decreased trabecular bone volume	Osteoblast-specific knockout of PTHrP results in reduced bone volume and changes in bone microstructure.	^227^

**Fig. 5:**
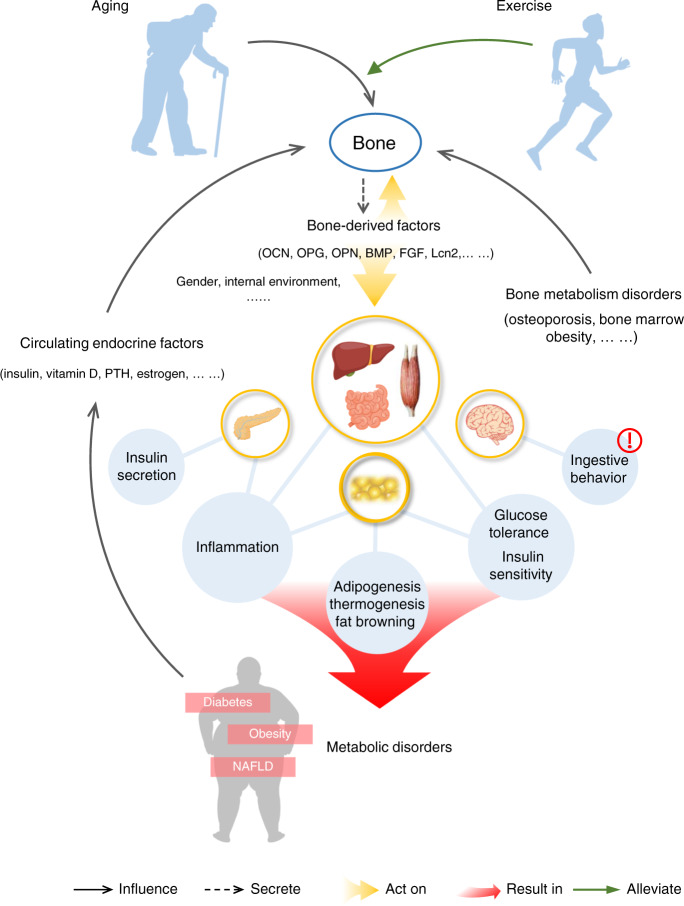
The network relationship between bone and whole-body energy metabolism. Aging is one of the main causes of bone and whole-body metabolism disorder, and exercise training can alleviate it. Changes in bone metabolism will affect multiple organs and tissues, including the brain (a potential research direction in the future), and lead to metabolic diseases. Metabolic diseases, as well as changes in circulating endocrine factors, can in turn affect bone. On the other hand, bone-derived factors can also be used as autocrine factors to regulate their own metabolic state

In view of the complex regulation of whole-body energy metabolism by bone, the clinical application of bone-derived factors will become one of the future research directions. For example, studying the role of bone-derived factors in aging, which is a severe problem facing society, may explain some diseases. In the process of human aging, bone metabolism and whole-body metabolism are continually changing. Aging may lead to metabolic disorders and affect bone metabolism. Macroscopically, the aging of bone is characterized by osteoporosis and a decrease in organic and inorganic components. At the same time, its significance is marked by a decrease in bone cells and the transformation of BMSCs into MAT and osteoblasts.^353,354^ Bone-derived factors from these cells are implicated in changes such as a decrease in glucose tolerance, the occurrence of insulin resistance, and the enhancement of the inflammatory response and may be caused by an imbalance in the levels of bone-derived factors, which leads to the occurrence of metabolic diseases. Bone is an important motor organ, so exercise can have a significant impact on bone metabolism.^355^ Not only bone but also exercise has been found to change the human body’s metabolic state and delay aging.^356,357^ One of the possible mechanisms is by affecting bone metabolism and the secretion of bone-derived factors. Therefore, exercise therapy may be used to treat or prevent metabolic diseases, especially those related to aging, in the future (Fig. 5).

The next question is whether we can use bone-derived factors as a marker for the diagnosis or as a new target for the treatment of metabolic diseases and their related complications. This conjecture has been verified by some research. For example, OPG has been found to be a potential marker for the diagnosis of diabetes in postmenopausal women,^358^ and experiments have explored whether regulating the proportion of OPG-RANKL-RANK signaling can interfere with the process of cardiovascular complications noted in patients with diabetes.^359^ More experiments are needed to further apply bone-derived factors to the treatment of clinical diseases.
